# Bidirectional Hebbian Plasticity Induced by Low-Frequency Stimulation in Basal Dendrites of Rat Barrel Cortex Layer 5 Pyramidal Neurons

**DOI:** 10.3389/fncel.2017.00008

**Published:** 2017-02-01

**Authors:** Andrea Díez-García, Natali Barros-Zulaica, Ángel Núñez, Washington Buño, David Fernández de Sevilla

**Affiliations:** ^1^Departamento de Anatomía, Histología y Neurociencia, Facultad de Medicina, Universidad Autónoma de MadridMadrid, Spain; ^2^Instituto Cajal, Consejo Superior de Investigaciones Científicas (CSIC)Madrid, Spain

**Keywords:** Ca^2+^spikes, NMDARs, L-type VGCC, dentritic excitabilty, STDP

## Abstract

According to Hebb's original hypothesis (Hebb, 1949), synapses are reinforced when presynaptic activity triggers postsynaptic firing, resulting in long-term potentiation (LTP) of synaptic efficacy. Long-term depression (LTD) is a use-dependent decrease in synaptic strength that is thought to be due to synaptic input causing a weak postsynaptic effect. Although the mechanisms that mediate long-term synaptic plasticity have been investigated for at least three decades not all question have as yet been answered. Therefore, we aimed at determining the mechanisms that generate LTP or LTD with the simplest possible protocol. Low-frequency stimulation of basal dendrite inputs in Layer 5 pyramidal neurons of the rat barrel cortex induces LTP. This stimulation triggered an EPSP, an action potential (AP) burst, and a Ca^2+^ spike. The same stimulation induced LTD following manipulations that reduced the Ca^2+^ spike and Ca^2+^ signal or the AP burst. Low-frequency whisker deflections induced similar bidirectional plasticity of action potential evoked responses in anesthetized rats. These results suggest that both *in vitro* and *in vivo* similar mechanisms regulate the balance between LTP and LTD. This simple induction form of bidirectional hebbian plasticity could be present in the natural conditions to regulate the detection, flow, and storage of sensorimotor information.

## Introduction

The rat somatosensory barrel field cortex (“barrel cortex”) processes sensorimotor information from the whiskers mainly through the thalamocortical inputs in Layers 4 and 5. Layer 5 (L5) pyramidal neurons (PNs), receiving a robust thalamocortical input at their basal dendrites that is weaker at their apical dendrites, produce the main output of the barrel cortex (Ramaswamy and Markram, 2015). The Ca^2+^-mediated dendritic spikes in L5 PNs are markedly reduced by inhibition of NMDA receptors (NMDAR) and L-type voltage gated Ca^2+^ channels (VGCCs) and have been called NMDA-spikes (Schiller et al., 2000; Polsky et al., 2009). Dendritic Ca^2+^ spikes play a leading role in the genesis of long-term potentiation (LTP) allowing a robust Ca^2+^ influx into PN spines (London and Hausser, 2005; Remy and Spruston, 2007). Under blockade of γ-aminobutyric acid type A receptors (GABA_A_Rs), regular spiking L5 PNs in the immature rat barrel cortex can trigger NMDA-spikes that cause a robust Ca^2+^ influx (Schiller and Schiller, 2001; Gordon et al., 2006; Polsky et al., 2009; Nuñez et al., 2012). An opposing form of synaptic plasticity is long-term depression (LTD) that is caused by a repeated synaptic input leading a small or local postsynaptic Ca^2+^ rise (Artola et al., 1990; Bliss and Collingridge, 1993; Neveu and Zucker, 1996; Dan and Poo, 2004; Holthoff et al., 2004; Kampa et al., 2007).

Importantly, long-term modifications in synaptic efficacy have been widely proposed to be the cellular basis of the learning machinery of the brain (Nabavi et al., 2014; Gruart et al., 2015). A physiologically relevant protocol for inducing “hebbian LTP” is spike-timing-dependent plasticity (STDP), which consists in repeatedly pairing at low-frequency an EPSP with postsynaptic action potentials (APs) induced by depolarizing current injection (Bi and Poo, 1998; Fuenzalida et al., 2007, 2010; Caporale and Dan, 2008; Feldman, 2012; Ramaswamy and Markram, 2015). However, Hebb's original postulate holds that synapses are potentiated when presynaptic activity triggers postsynaptic firing (Hebb, 1949) and it does not predict the necessity of pairing presynaptic and postsynaptic stimulation. Nevertheless, the underlying cellular and network mechanisms required to trigger either LTP or LTD with this simpler form of “unpaired” low-frequency presynaptic stimulation remain unclear. The term unpaired is used to indicate that under current-clamp we stimulate the afferent pathway without manipulating the postsynaptic neuron. A different form of long term response enhancement induced by unpaired low-frequency stimulation of tuft dendrite inputs that is only expressed in L5 PN tuft dendrites in disinhibited slices has recently been reported (Sandler et al., 2016). In addition, we have shown that acetylcholine can facilitate long-term response enhancement induced by low frequency stimulation of basal inputs in L5 PNs (Nuñez et al., 2012).

Therefore, we analyzed *in vitro* the underlying cellular and network mechanisms required to trigger either LTP or LTD with the simpler form of unpaired low-frequency presynaptic stimulation. We show that *in vitro* under GABA_A_R blockade, a robust LTP could be induced by low-frequency stimulation of L5 PN basal synaptic inputs (termed hereafter “basal stimulation”) evoking an EPSP followed by an AP burst and a Ca^2+^ spike (termed hereafter “EPSP-Ca^2+^ spike”). The resulting LTP required Ca^2+^ influx through NMDARs and L-type VGCC, Ca^2+^ release from intracellular stores and activation of glutamatergic subtype I, muscarinic subtype 3, and nicotinic ACh receptors. The contribution of Ca^2+^/calmodulin-dependent protein kinase II (CaMKII), phospholipase C (PLC), and protein kinase A (PKA) were also necessary. Inhibition of NMDARs, L-type VGCCs, or membrane hyperpolarization could reduce the EPSP-Ca^2+^ spikes and the associated Ca^2+^ signal and induce LTD instead of LTP. Blockade of voltage gated Na^+^ channels also induced LTD in place of LTP. Consequently, it was possible to regulate the sign of the induced change in synaptic plasticity using the level of membrane depolarization attained during the Ca^2+^ spike. Importantly, basal stimulation could trigger APs despite intact inhibition but failed to evoke Ca^2+^ spikes and plasticity. We also show *in vivo* in anesthetized rats that repeated low-frequency whisker deflections can induce a similar NMDAR-dependent bidirectional plasticity, suggesting a causal relationship between network function and sensory detection.

Overall, low-frequency stimulation of basal inputs and whisker deflections can induce forms of bidirectional plasticity, possibly through the regulation of dendritic excitability in L5 barrel cortex neurons, caused by a reduced GABA_A_ inhibition that could be functional in the natural situation and regulate both the flow and storage of novel input characteristics and the balance between LTP and LTD.

## Materials and methods

### Ethical approval and animal handling

Procedures of animal care and slice preparation approved by the “Universidad Autónoma de Madrid” and “Consejo Superior de Investigaciones Científicas” follow the guidelines laid down by the European Council on the ethical use of animals (Directive 2010/63/EU) and every effort was made to minimize animal suffering and number. The procedures have been described in detail elsewhere (Nuñez et al., 2012).

### *In Vitro* experiments

#### Slice preparation and drug applications

**Figure 1:**
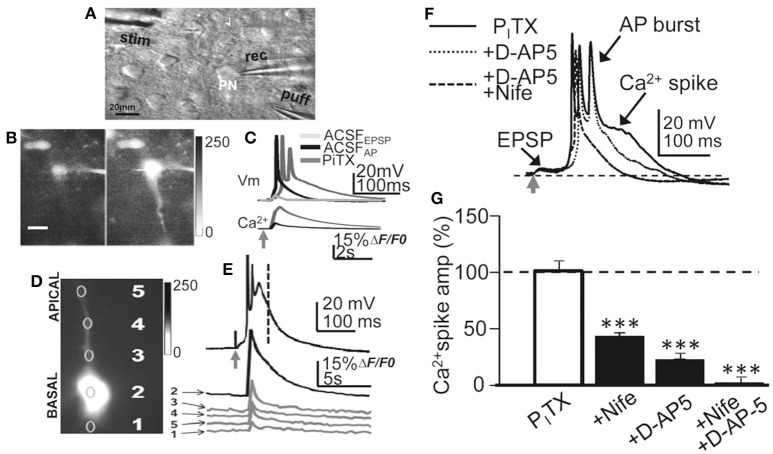
**Experimental setup, current-clamp responses and Ca^**2+**^ signals**. **(A)** DIC image of a slice showing a L5 PN and placement of recording (rec) stimulation (stim) and glutamate (puff) pipettes. **(B)** left. Representative image (gray scale) showing a small somatic Ca^2+^ elevation evoked by basal stimulation that triggered an EPSP and a single AP in control ACSF. **(B)** right. Same as left but a larger Ca^2+^ signal in the soma and dendrites under the P_I_TX (50 μM) that triggered an EPSP-Ca^2+^ spike (**A** and **B**, same PN). **(C)**, upper. Representative superimposed records showing EPSP, EPSP+AP in control ACSF and the EPSP-Ca^2+^ spike under P_I_TX. **(C)**, lower. Time course of somatic cytosolic Ca^2+^ variations during EPSC+AP and EPSP-Ca^2+^ spike in **(B)**, left. **(D)** Somatic (2), basal (1) and apical (3-5) dendritic cytosolic Ca^2+^ variations associated with the averaged EPSP-Ca^2^ spike in **(E)**. **(E)** Current-clamp response under P_I_TX (upper) and Ca^2+^ signals obtained from specified regions of interest (1–5 in **D**). **(F)** Representative superimposed records obtained under P_I_TX and after adding D-AP5 and D-AP5+Nifedipine. **(G)**. Bar plot showing the effects of nifedipine (20 μM, *N* = 10, *P* < 0.001), D-AP5 (50 μM, *N* = 6, *P* < 0.001) and nifedipine+D-AP5 (*N* = 9, *P* < 0.001) on the amplitude of the Ca^2+^ spike measured at delays indicated by the vertical interrupted line in **(E)**.

Young Sprague Dawley rats (12–19 days old) of either sex were decapitated, and their brains were removed and submerged in cold (≈ 4° C) solution (in mM): Choline-Cl 120.00; KCl 2.50; KH_2_PO_4_ 1.25; Mg_2_SO_4_ 2.00; NaHCO_3_ 26.00; CaCl_2_ 2.00; Na^+^ aspartate 3.00; and Ascorbic acid 0.40. pH was stabilized at 7.4 by bubbling the solution with carbogen (95% O_2_, 5% CO_2_). Transverse slices (400 μm) containing the barrel cortex were cut with a Vibratome (Pelco 3000, St Louis, USA or Leica VT 1200S) and incubated >1 h in control artificial cerebro-spinal fluid (ACSF) at a room temperature of 20–22°C. The ACSF contained (in mM): 124.00 NaCl, 2.69 KCl, 1.25 KH_2_PO_4_, 2.00 Mg_2_SO_4_, 26.00 NaHCO_3_, 2.00 CaCl_2_, and 10.00 glucose. Slices were placed in a 2 ml chamber fixed to an upright microscope stage (BX51WI; Olympus, Tokyo, Japan) equipped with infrared differential interference contrast video (DIC) microscopy and a 40X water-immersion objective (Figure 1A). Slices were superfused with carbogen-bubbled ACSF (2 ml/min) and maintained at room temperature. Picrotoxin (P_I_TX, 50 μM), D-2-amino-5 phosphonovaleric acid (D-AP5; 50 μM) and 7-nitro-2,3-dioxo-1,4-dihydroquinoxaline-6-carbonitrile (CNQX; 20 μM) were used to isolate the EPSCs. (S)-α-Methyl-4-carboxyphenylglycine(+)-alpha-methyl-4-carboxyphenylglycine (MCPG; 1.0 mM); 2-Methyl-6-(phenylethynyl) pyridine hydrochloride (MPEP; 5.0 μM); (S)-(+)-α-Amino-4-carboxy-2-methylbenzeneacetic acid (LY367385; 50 μM) were used as required. Atropine (0.3 μM), pirenzepine (75 nM), methoctramine (1 μM), Mecamylamine (MMA) (10 μM), methyllycaconitine (MLA, 50 μM), and α7-containing neuronal nAChR antagonist were also used. Nifedipine (20 μM), DAU5884 hydrochloride (1 μM), U73122 (5 μM) and H89 dihydrochloride (10 μM) were dissolved in DMSO (0.01%) and added to the ACSF as needed. Chemicals were purchased from Sigma-Aldrich Quimica (Madrid, Spain), Tocris Bioscience (Ellisville, MO; distributed by Biogen Cientıfica, Madrid, Spain), and Alomone Labs (Jerusalem, Israel). Brief localized “puffs” of glutamate (1 mM, 100–300 ms duration 2.0–2.5 *psi*) were applied through a pipette (tip diameter ≈ 5 μm) connected to a Picospritzer II (General Valve, Fairfield, NJ) and placed near the basal dendrites (50–100 μm) of the recorded L5 PN with a hydraulic micromanipulator (Narishige, Tokyo, Japan).

#### Electrophysiology

Whole-cell patch-clamp recordings were obtained from the soma of L5 PNs using patch pipettes (4–8 MΩ) filled with a solution that contained (in mM): 135 K-gluconate, 10.0 HEPES, 0.2 EGTA, 2.0 Na_2_-ATP, and 0.4 Na_3_-GTP, buffered to pH 7.2–7.3 with KOH. Intracellular solutions could also contain either 1,2-Bis (2-aminophenoxy) ethane-N,N,N, N'-tetraacetic acid (BAPTA; 40 mM), heparin sodium salt (5.0 mg/ml), ruthenium red (Ru-Red; 400 μM), AIP (Autocamtide 2-related inhibitor peptide; 5 mg/ml), GDPβS (1 mM), the quaternary lidocaine derivative QX-314 (5 mM), or chelerytrine (5 mM). Recordings were performed in current- or voltage-clamp modes using a Cornerstone PC-ONE amplifier (DAGAN, Minneapolis, MN). Pipettes were set in place with a mechanical micromanipulator (Narishige). The holding potential was adjusted to −60 mV, and the series resistance was compensated to ≈ 80%. L5 PNs located beneath the barrels were only accepted if the seal resistance was >1 GΩ before breaking into whole cell and the series resistance (7–14 MΩ) did not change >15%, and the holding current did not exceed 300 pA at −75 mV during the experiment. The junction potential (≈6 mV) was not corrected. Data were low-pass filtered at 3.0 kHz and sampled at 10.0 kHz, through a Digidata 1322A (Molecular Devices, Sunnyvale, CA) with the pClamp programs (Molecular Devices).

#### Synaptic stimulation

Bipolar stimulation used either a concentric electrode (OP 200 μm, IP 50 μm, FHC) or a pipette pulled from theta glass capillary (Ø of the tip ≈ 20–40 μm), filled with ACSF and connected though two silver-chloride wires. A Grass S88 stimulator and stimulus isolation unit (Quincy, USA) was used and no significantly different results were observed with the two electrodes. Electrodes were placed 50–100 μm below the soma of the patched PN. Single pulses were continuously delivered at 0.3 Hz. After a 5 min control recording of EPSCs, the recording was switched to current-clamp, and stimulation intensity was increased to values in which the EPSP triggered APs and Ca^2+^ spikes. This stimulation was applied 60 times at 0.2 Hz, the recording was then switched back to voltage-clamp and stimulation intensity and frequency restored to the initial control values. We decided to stimulate at 0.2 Hz because frequencies >0.5 Hz caused frequent Ca^2+^ spike failures and did not induce LTP; frequencies <0.1 Hz were less effective in inducing LTP. However, the precise stimulation frequency requirements for the induction of this LTP still remain to be determined. The pre- or postsynaptic origin of EPSC amplitude change was investigated by computing the PPR and the EPSC variance that parallels the changes in EPSC amplitude. Paired pulses (100 ms interval) were used in a group of experiments to determine changes in presynaptic release probability by computing a paired-pulse response ratio (PPR) as the quotient of the second EPSC of the pair over the first EPSC (R2/R1). PPRs above and below one respectively corresponded to paired-pulse facilitation (PPF) or paired-pulse depression (PPD), indicating the respective low and high release probabilities. To estimate the EPSC variance modifications, we first calculated the noise-free coefficient of variation (CV_NF_) of the synaptic responses in control conditions and then ≈ 30 min after the induction of the Ca^2+^ spikes. We used the formula CV_NF_ = √($\delta_{\text{EPSC}}^{2}$ − $\delta_{\text{noise}}^{2}$)/*m*, where $\delta_{\text{EPSC}}^{2}$ and $\delta_{\text{noise}}^{2}$ are the variances of the peak EPSC and the baseline, respectively, and *m* is the mean EPSC peak amplitude. The ratio of the CV (CVR) measured at ≈ 30 min over that in control conditions was obtained for each neuron as CV_afterCaSpikes_/CV_control_ (Fernández de Sevilla et al., 2002). Finally, we constructed plots comparing variation in average EPSC amplitude (M) with the change in response variance of the EPSC amplitude (1/CVR^2^) in each cell (Fernández de Sevilla et al., 2002). Values, in these plots, should follow the diagonal if the EPSC potentiation has a presynaptic origin. This method requires a binomial EPSC amplitude distribution but we could not directly test whether our data fitted the binomial distribution. Nevertheless, synaptic fluctuations were always evident and we assumed that synaptic release followed a binomial distribution.

#### Calcium imaging

Simultaneous electrophysiology and cytosolic Ca^2+^ imaging were obtained by filling patch pipettes with a solution containing 50–100 μM fluo-3 (Molecular Probes, Eugene, OR, USA). Imaging experiments were performed after a 10–15 min stabilization period that allowed equilibration of the dye. Slices were illuminated for 40 ms every 200 ms at 490 nm with a monochromator (Polychrome IV; TILL Photonics) and successive images were obtained at 5 s^−1^ with a cooled monochrome CCD camera (Luca, Andor Technologies) attached to the Olympus microscope equipped with a filter cube optimized for fluo-3. Camera control, synchronization with electrophysiological measurements and quantitative epifluorescence measurements were made with the ImagingWorkbench software (INDEC-BioSystems, Santa Clara, CA, USA). Fluctuations in fluorescence were expressed as the proportion (%) of relative change in fluorescence (Δ*F/F*_*0*_) where *F*_*0*_ is the pre-stimulus fluorescence level when the cell is at rest and Δ*F* is the change in fluorescence during activity. Plots of Ca^2+^ signal variations vs. time were obtained “off-line” at specified regions of interest from stored image stacks and expressed as Δ*F/F*_*0*_. Corrections were made for indicator bleaching during trials by subtracting the signal measured under the same conditions when cells were not stimulated. Although, we could record the strong calcium signal associated to the Ca^2+^ spikes (Figures 1B,C), the low temporal resolution of our Ca^2+^ recordings did not allow us to resolve the site of initiation of the Ca^2+^ signal (Figures 1D,E), although it probably originated at the basal dendrites and propagated rapidly to the soma and apical dendrite.

#### Data analysis

Data were analyzed with the pClamp programs (Molecular Devices, Chicago, USA) and Excel (Microsoft, Redmond, USA) and responses were averaged (*n* = 10 or 20), except when indicated otherwise. The magnitude of the change in peak amplitude of EPSCs was expressed as a proportion (%) of the baseline control amplitude and plotted in function of time. The amplitude and duration of the Ca^2+^ spikes was measured after the AP burst had ended (50 ms) and when the membrane potential reached pre-stimulation values (300 ms). Results are given as average ± SEM (*N* = number of cells), and presented as percentage of controls. Statistical analyses were calculated with Student's two-tailed *t*-tests for unpaired or paired data as required. The threshold for statistical significance was *P* < 0.05 (^*^); *P* < 0.01 (^**^); and *P* < 0.001(^***^) are also indicated. Gender related differences were not detected in our sample.

### *In vivo* experiments

#### Electrophysiological recordings

Experiments were performed on 18 urethane-anesthetized (1.6 g/kg i.p.) Sprague Dawley rats weighing 200–250 g. Animals were placed in a Kopf stereotaxic device, the body temperature was maintained at 37°C, and the end-tidal CO_2_ and heart rate were monitored. Lidocaine (1%) was applied to all skin incisions and additional doses of anesthetic were delivered to maintain areflexia. An incision was made exposing the skull and a small hole was drilled in the bone over the barrel cortex (A 1–3 mm, L 5–7 mm from bregma). Single-unit recordings in L5 barrel cortex were made 900–1200 μm below the surface with tungsten microelectrodes (2–5 MΩ). Recordings were filtered (0.3–3.0 kHz), amplified *via* an AC preamplifier (DAM80; World Precision Instruments), and fed into a personal computer (sample rate 10.0 kHz) together with the temporal references of the stimuli for off-line analysis with Spike 2 software (Cambridge Electronic Design, Cambridge, UK).

#### Whisker stimulation and induction of plasticity

Whisker deflections were generated by brief 20 ms air puffs using a pneumatic pressure pump (Picospritzer) delivered through a 1-mm-inner diameter polyethylene tube (**Figure 8A**). The air pressure was set at 1–2 kg/cm^2^, resulting in whisker deflections of ≈ 15°. When a single neuron was isolated, its cutaneous receptive field was carefully mapped with a small hand-held brush and the response of the principal whisker was confirmed. The protocol used to investigate plasticity consisted of 30 air pulses delivered to the principal whisker at 0.5 Hz (CONTROL), followed by a train of 40 pulses at 1.0 Hz (INDUCTION) delivered to the same whisker. Thirty pulses at 0.5 Hz were then delivered to the same whisker 1, 5, 15, and 30 min after (POST-INDUCTION). This stimulation protocol could either induce LTP or depression (LTD) of whisker-evoked AP responses (see below). In some experiments NMDARs were inhibited with D-AP5 (50 μM; 0.1 μl) injected through a cannula connected to a 5 μl Hamilton syringe and targeted on L5. Five minutes after the injection the complete experimental protocol began: control, induction train, and post-induction whisker stimulations during 30 min.

#### Data analysis

Peristimulus time histograms (PSTH; 1 ms bin width; 30 successive stimuli) were computed during the CONTROL, INDUCTION, and POST-INDUCTION periods. Whisker-evoked responses were estimated from the total number of spikes evoked over a 100 ms post-stimulation time window, divided by the number of stimuli. To determine differences in whisker-evoked responses induced by the experimental manipulations, cumulative spike plots (CSPs) were constructed by adding successive PSTH bins. A grand average of all PSTHs computed over a given experimental condition was also constructed, and the corresponding averaged CSPs were computed.

## Results

### Basal stimulation triggered EPSP-Ca^2+^ spikes in disinhibited slices

**Figure 2:**
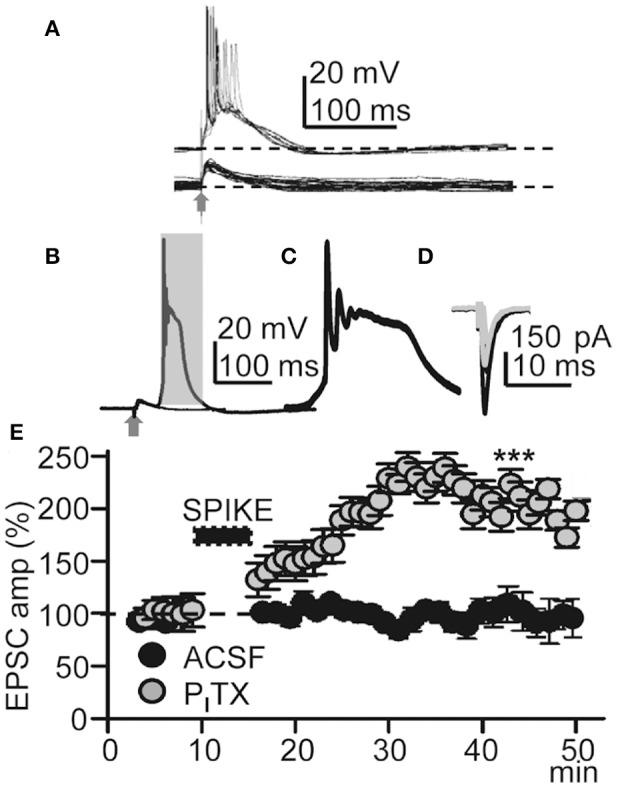
**Repeated Ca^**2+**^ spikes induce LTP**. **(A)** Representative superimposed EPSPs recorded in control ACSF (bottom) and superimposed EPSP-Ca^2+^ spikes (*n* = 20) recorded under P_I_TX (50 μM) (top); note AP bursts. **(B)** Superimposed averaged EPSP and EPSP-Ca^2+^ spike (*n* = 20 as in all other Figures) recorded under P_I_TX. **(C)** Expanded time record taken from the shaded area in **(B)**. **(D)** Representative superimposed averaged EPSCs recorded before and ≈30 min after LTP induction (gray and black records, respectively). **(E)** Time course of the peak EPSC amplitude (% of controls, as in all other Figs.) in ACSF (black circles, *N* = 8, *P* > 0.05) and under P_I_TX (gray circles, *N* = 10, *P* < 0.001).

Recordings were obtained from slender tufted L5A PNs (*N* = 244) and confirmed by intracellular biocytin staining (Nuñez et al., 2012). In control ACSF, basal stimulation induced an EPSP that above a threshold depolarization level triggered a single AP (Figure 1C). Increasing stimulation intensity could increase the number of APs (1–3 APs, with frequent failures) without further modifying the response. In contrast, under P_I_TX (50 μM) blockade of GABA_A_ inhibition, the EPSP could trigger at short delays of 5 ± 3 ms 1 AP or a brief high frequency burst of 2 and occasionally 3 APs with failures, that lasted 20 ± 8 ms (*N* = 14). The AP burst rode on a slow high amplitude depolarization wave (peak amplitude 53 ± 6 mV; duration 375 ± 60 ms; same cells; Figures 1C–F, 2A–C). The slow depolarization wave was not modified when stimulation intensity was further increased, displaying an all-or-none behavior.

The amplitude and duration of the slow depolarization wave was markedly reduced when either nifedipine (20 μM; reaching values of 47 ± 7% of the controls; *P* < 0.001; *N* = 10) or D-AP5 were superfused (50 μM; reaching values of 28 ± 5% of the controls; *P* < 0.001; *N* = 6) (Schiller and Schiller, 2001; Nuñez et al., 2012). When both D-AP5 and nifedipine were superfused the slow depolarization wave was totally suppressed (*P* < 0.001; *N* = 9; Figures 1F,G). In addition, a robust cytosolic Ca^2+^ signal that could be recorded in the soma, where the Δ*F/F*_*0*_ reached values that were 52 ± 9% of the controls (*P* < 0.001; *N* = 6), while in basal and apical dendrites, the Δ*F/F*_*0*_ attained values that were 19 ± 9% of the controls (*P* < 0.001; same cells; Figures 1B–E). Much smaller Ca^2+^ signals (8 ± 1% of controls, *P* < 0.01; same cells) were induced when a single AP was triggered in the absence of Ca^2+^ spikes (Figures 1B,C). The all-or-none behavior of the slow depolarization wave following EPSPs and the robust dendritic Ca^2+^ signals suggests that the slow depolarizations were Ca^2+^ spikes.

### EPSP-Ca^2+^ spikes triggered by low-frequency stimulation induced LTP

We first tested if EPSP-Ca^2+^ spikes triggered by low-frequency basal stimulation could induce LTP. Under blockade of GABA_A_Rs with P_I_TX (50 μM), basal stimulation induced inward EPSCs at −65 mV with mean peak amplitudes of −160 ± 9 pA (*P* < 0.001; *N* = 6). After a 5–10 min control recording of EPSCs under voltage-clamp with stimulation at 0.3 Hz, the recording was switched to current-clamp and stimulation intensity was increased until EPSP-Ca^2+^ spikes were evoked without failure. In these conditions the delay between the EPSP and the AP burst-Ca^2+^ spike was fixed for a given experiment although it could fluctuate between ≈ 2 and ≈ 20 ms in different experiments. EPSP-Ca^2+^ spikes were applied 60 times at 0.2 Hz, the recording was switched back to voltage-clamp, and stimulation intensity and frequency restored to the initial control conditions. The repeated EPSP-Ca^2+^ spikes induced a robust LTP typified by an increase in the mean peak EPSC amplitude that in ≈ 30 min reached values that were 210 ± 45% of the control (*P* < 0.001; *N* = 23; Figures 2D,E).

We also tested if the same basal stimulation protocol could induce plasticity when synaptic inhibition was active in control ACSF. In control ACSF Ca^2+^ spikes were never evoked by repeated basal stimulation and EPSC amplitudes were essentially identical to the controls, reaching values that were 97 ± 2% of the control (*P* > 0.05; *N* = 8; Figure 2E) in ≈ 30 min. The above results suggest that this LTP was intimately dependent on active dendritic mechanisms under close control by GABA_A_ inhibition (Wigström and Gustafsson, 1983; Kampa et al., 2007; Marlin and Carter, 2014).

**Figure 3:**
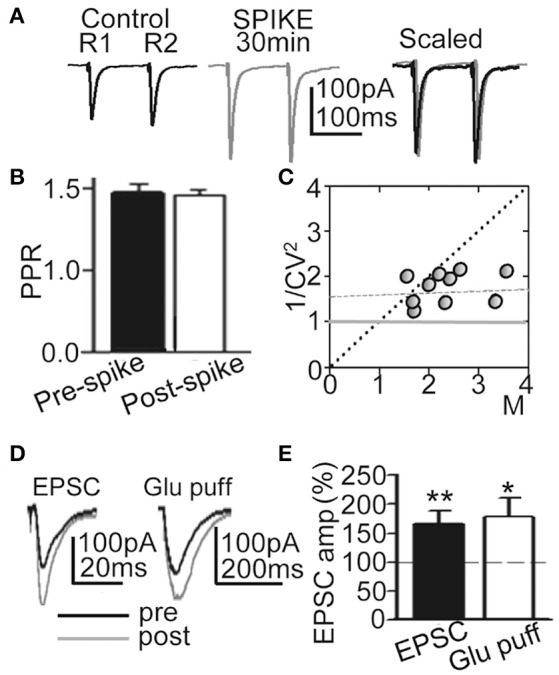
**LTP was induced without changes in Glu release probability**. **(A)** Representative averaged EPSCs (*n* = 20) evoked by paired pulse stimulation (100 ms delay) before (Control R1, R2), at ≈30 min after 60 EPSP-Ca^2+^ spikes (SPIKE, 30 min) and superimposed and scaled to the amplitude of the first ESPC (Scaled). **(B)** Summary data showing the unchanged PPR (% of control, *N* = 15, *P* > 0.05) before (Pre-spike) and ≈30 min after induction with 60 EPSP-Ca^2+^ spikes (Post-spike). **(C)** Plot of the variance (1/CV^2^) in function of the mean peak EPSC amplitude normalized to controls (M) measured ≈30 min after 60 EPSP-Ca^2+^ spikes were applied (*N* = 10). **(D)** left. Representative superimposed EPSCs recorded before and at ≈30 min after LTP induction. **(D)** right. Same as left, but currents evoked by Glu puffs. **(E)** Summary data showing changes in peak EPSC (*N* = 6, *P* < 0.01) and Glu puff current amplitudes (same cells, *P* < 0.05) 30 min after LTP induction, relative to control (100%).

To verify the pre- or postsynaptic origin of this LTP we first tested for possible increases in release probability at excitatory synapses. There were no modifications in PPR or the 1/CV^2^ ratio (Figures 3A–C), suggesting that this LTP was not associated with changes in the probability of glutamate (Glu) release, and that there was no presynaptic contribution to it. In addition, both EPSCs and currents evoked by Glu puffs (that bypass the presynaptic components of Glu transmission) were potentiated to similar values (166.5 ± 22.3%; *P* < 0.01; *N* = 6 and 169.2 ± 23.7, *P* < 0.05; same cells, for the EPSCs and Glu currents, respectively) following the induction of LTP (Figures 3D,E).

### Both the action potential burst and Ca^2+^ spike were required to induce this LTP

**Figure 4:**
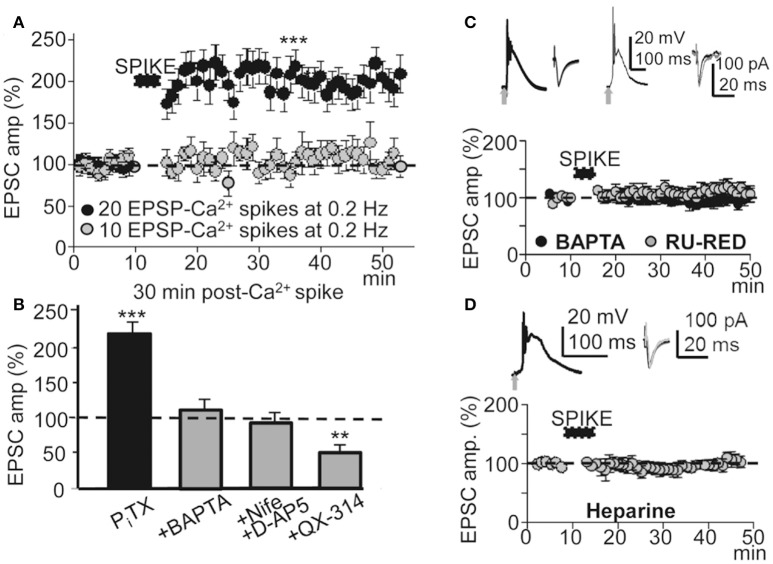
**A threshold number of EPSP-Ca^**2+**^ spikes was required to induce this LTP. Effects of blocking the Ca^2+^ spike, AP burst and Ca^2+^ release. (A)** Time course of the peak EPSC amplitude showing the effects of 10 (gray circles, *N* = 8, *P* > 0.05) and 20 successive EPSP-Ca^2+^ spikes (black circles, *N* = 6, *P* > 0.001), both at 0.2 Hz. **(B)** Bar plot showing average peak EPSC amplitudes relative to pre-induction controls (100%) under P_I_TX (50 μM, *N* = 10, *P* < 0.001) and the effects of antagonizing the cytosolic Ca^2+^ rise by chelation with +BAPTA-loading (40 mM in the pipette solution, *N* = 6, *P* > 0.05), of inhibition of the Ca^2+^ spike by superfusing with nifedipine (20 μM) + D-AP5 (50 μM) (+Nife+D-AP5, *N* = 9, *P* > 0.05) and of blocking voltage dependent Na^+^ currents with intracellular +QX-314 (5 mM in the pipette solution, *N* = 6, *P* > 0.05). **(C)** upper left. Representative averaged EPSP-Ca^2+^ spike recorded in a BAPTA-loaded PN (40 mM in the intracellular solution) and superimposed EPSCs recorded before and ≈30 min after LTP induction. **(C)** upper right. Same as left, but in a RU-RED-loaded cell (400 μM in the intracellular solution). **(C)** bottom. Time course of the peak EPSC amplitude in BAPTA-loaded (black circles, *N* = 6, *P* > 0.05) and RU-RED-loaded (gray circles, *N* = 6, *P* > 0.05) PNs. **(D)** same as **(A)**, but in Heparin-loaded PNs (5 mg/ml, *N* = 6, *P* > 0.05).

To determine the contribution of the Ca^2+^ spike to the induction of this LTP under P_I_TX (50 μM), we inhibited Ca^2+^ spikes through a blockade of NMDARs with D-AP5 (50 μM), and of L-type VGCCs with nifedipine (20 μM). Basal stimulation was increased well above the intensity (x2) at which the EPSP triggered APs to compensate for the EPSP amplitude reduction caused by the blockade of the NMDA component. In these conditions the Ca^2+^ spike was blocked (Figures 1F,G) and basal stimulation (60 times at 0.2 Hz) was unsuccessful in inducing LTP (EPSCs reached values that were 98 ± 9% of the controls, *P* > 0.05; *N* = 6; Figure 4B).

In L5 PNs STDP requires pairing an EPSP with an AP burst induced by depolarizing current injection to rescue AP back-propagation through the generation of a Ca^2+^ spike (Larkum et al., 1999b; Kampa et al., 2004). Accordingly, we tested the effects of avoiding the AP burst by antagonizing voltage-gated Na^+^ channels with intracellular QX-314 under PITX (50 μM). Under QX-314-loading (5 mM in the pipette solution) APs were inhibited and the EPSP and Ca^2+^ spike remained (see below). In addition, under QX-314 the peak amplitude of Ca^2+^ spikes (58 ± 5 mV, *N* = 6) were even larger than those linked with the induction of LTP (53 ± 6 mV, see above), likely indicating that what was required for LTP was not just the Ca^2+^ rise, but that Na^+^-mediated back-propagating APs played a key role. In these conditions, repeated basal stimulation (60 times at 0.2 Hz) induced a robust LTD instead of LTP, and EPSCs reached values that were 48 ± 6% of the controls (*P* < 0.01; *N* = 6) ≈ 30 min after the onset of stimulation (Figure 4B). These results suggest that the AP burst and Ca^2+^ spike were essential to the induction of this LTP.

### A cytosolic Ca^2+^ rise is a prerequisite for the induction of this LTP

To determinate the contribution of the cytosolic Ca^2+^ rise in the LTP induction, we tested the effects of BAPTA-loading, which chelates Ca^2+^, preventing a rise of Ca^2+^ in the cytosol. BAPTA-loading (40 mM in the pipette solution) did not modify the EPSP-Ca^2+^ spike (Figure 4C), and repeated basal stimulation (60 times at 0.2 Hz) was unable to induce LTP while EPSCs reached values that were 103 ± 6% of the controls (*P* > 0.05; *N* = 6) ≈ 30 min after the induction process (Figures 4B,C).

Because Ca^2+^ release from IP3-sensitive stores and Ca^2+^ induced-Ca^2+^ release (CICR) through ryanodine receptors can contribute to the cytosolic Ca^2+^ rise we tested the effects of blocking the ryanodine receptors with intracellular ruthenium red (Ru-Red 400 μM in the pipette solution). Ru-Red blocked the LTP without modifying the EPSP-Ca^2+^ spike, while EPSCs reached values that were 99 ± 11% of the controls (*P* > 0.05; *N* = 6; Figure 4C) ≈ 30 min after the induction process. We next examined the effects of blocking IP_3_Rs with intracellular heparin (5 mg/ml in the pipette solution). In these conditions the LTP was inhibited without modification of the EPSP-Ca^2+^ spike and the EPSCs reached amplitudes that were 86 ± 8% of the controls (*P* > 0.05 *N* = 6) ≈ 30 min after 60 basal stimulations at 0.2 Hz (Figure 4D). Therefore, a rise in cytosolic Ca^2+^ was required for the induction of this LTP and was produced by influx through NMDARs, L-type VGCC and release from intracellular stores.

### This LTP required G-proteins, activation of metabotropic glu receptors, and muscarinic and nicotinic AChRs

**Figure 5:**
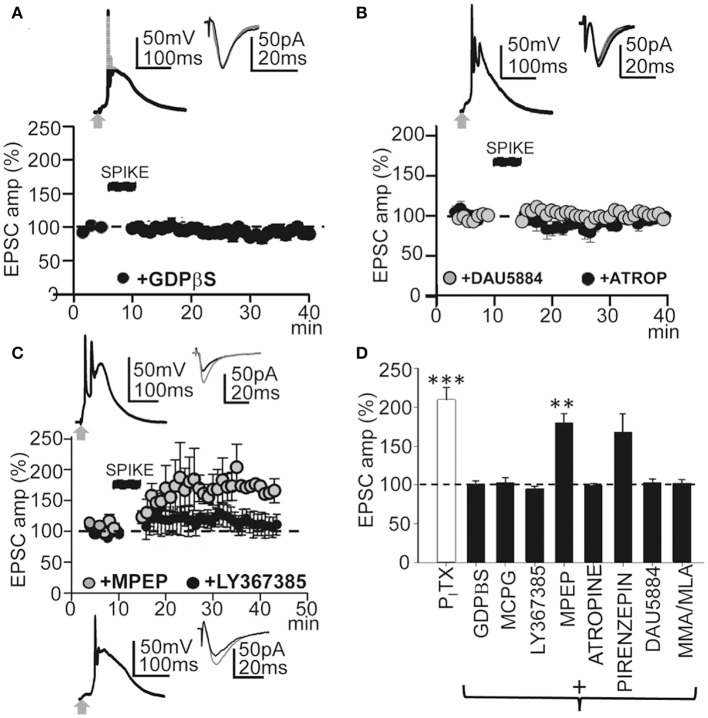
**GPCRs and metabotropic receptors participate in the induction of this LTP. (A)** upper. Averaged EPSP-Ca^2+^ spike and superimposed EPSCs recorded with intracellular GDPβS (1 mM in the pipette solution). **(A)** lower. Time course of the peak EPSC amplitude showing the effects of intracellular GDPβS (*N* = 6, *P* > 0.05). **(B)** Same as in **(A)**, but effects of superfusion with atropine (ATRO, 0.3 μM; filled circles, *N* = 5, *P* > 0.05) and DAU5884 (1 μM, gray circles, *N* = 6, *P* > 0.05). **(C)** Same as in **(A)**, but effects of superfusion with LY367385 (50 μM, top insets and black circles, *N* = 5, *P* > 0.05) and with MPEP (5.0 μM, gray circles and bottom insets, *N* = 6, *P* < 0.001). **(D)** Summary data showing the effect of P_I_TX (*N* = 10, *P* < 0.001), GDPβS (*N* = 6, *P* > 0.05), MCPG (1.0 mM, *N* = 4, *P* > 0.05), LY367385 (*N* = 5, *P* > 0.05), MPEP (*N* = 6, *P* < 0.01), atropine (*N* = 5, *P* > 0.05), Pirenzepine (75 nM, *N* = 5, *P* < 0.01), DAU5884 (*N* = 6, *P* > 0.05) and MMA/MLA (10/50 μM, *N* = 5, *P* > 0.05), relative to EPSCs in control ACSF (100%). 60 EPSP-Ca^2+^ spikes were used in **(A–D)**.

Synaptic plasticity is controlled by intracellular cascades in which G-protein coupled receptors (GPCRs) play key roles (Mukherjee and Manahan-Vaughan, 2013). Therefore, we first tested the effects of blocking G-proteins by loading the PN with GDPβS. With intracellular GDPβs (1 mM in the pipette solution) EPSP-Ca^2+^ spikes continued but failed to induce LTP. Thirty min after the induction protocol, EPSCs reached values that were 101 ± 6% of the controls (*P* > 0.05; *N* = 6; Figures 5A,D). Since metabotropic receptors are coupled to G-proteins we checked whether metabotropic Glu receptors (mGluRs) were involved in LTP induction. Superfusion with MCPG (1.0 mM), a group I/II mGluR antagonist, prevented LTP and, ≈ 30 min after induction, EPSCs reached values that were 92 ± 25% of the controls (*P* > 0.05; *N* = 4, Figure 5D). Although there was a small but not significant increase in EPSC amplitude, there was no LTP with the selective mGluR1 antagonist LY367385 (50 μM) and EPSC amplitudes reached values that were 109 ± 25% of the controls (*P* > 0.05; *N* = 5; Figures 5C,D). In contrast, a robust LTP was induced ≈30 min after the EPSP-Ca^2+^ spike in the presence of the mGluR5 selective antagonist MPEP (5.0 μM), with EPSC amplitudes that reached 196 ± 32% of the controls (*P* < 0.01; *N* = 6; Figures 5C,D). Therefore, mGLuR1 activation was required to induce the LTP.

Muscarinic AChRs (mAChRs) play a key role in certain forms of long-term enhancement of excitatory synaptic transmission (Fernández de Sevilla et al., 2008; Buchanan et al., 2010; Fernández de Sevilla and Buño, 2010; Nuñez et al., 2012; Dennis et al., 2016). Accordingly, we tested the effects of the non-selective mAChR antagonist atropine (0.3 μM), which prevented LTP induction. Under atropine EPSCs reached values that were 101 ± 2% of the controls (*P* > 0.05; *N* = 5) ≈ 30 min after the induction process (Figure 5D). DAU5884 (1 μM), a selective subtype 3 mAChR antagonist, blocked the LTP and EPSCs reached values of 103 ± 4% of the controls (*P* > 0.05; *N* = 5) ≈ 30 min after induction (Figures 5B,D). Pirenzepine (75 nM), a selective M1 mAChR antagonist, did not prevent the LTP and EPSCs reached values of 168 ± 5% of the controls (*P* < 0.01; *N* = 5) ≈30 min after induction (Figure 5D). Nicotinic AChRs (nAChRs) have also been involved in the regulation of transmitter release and synaptic plasticity. Therefore, we tested the effects of blockade of α4β2-containing and α7-containing nAChRs using MMA (10 μM) plus MLA (50 μM). This prevented LTP induction and EPSC reached values that were 102 ± 4% of the controls (*P* > 0.05; *N* = 5; Figure 5D). Taken together these results suggest that this LTP required the participation of GPCRs and activation of mGluR R1 and subtype M3 mAChRs, as well as nAChRs. Note that none of these treatments modified the EPSP-Ca^2+^ spikes, suggesting that the inhibition of LTP occurred downstream of the cytosolic Ca^2+^ rise.

### LTP induction required kinase activation

**Figure 6:**
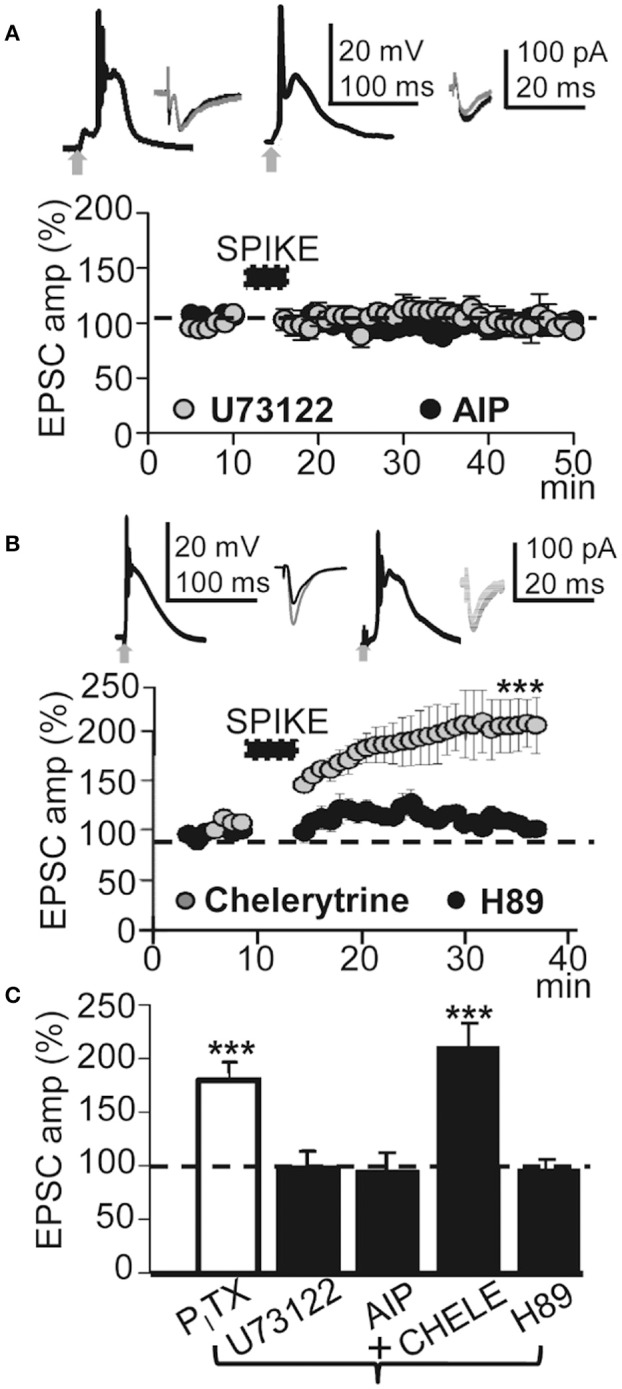
**This LTP required activation of PLC and PKA. (A)** , upper left. Averaged EPSP-Ca^2+^ spike recorded with intracellular U73122 (2 mM in the pipette solution). **(A)**, upper right. Same as left but intracellular AIP. **(A)**, lower. Time course of the peak EPSC amplitude showing the effects of intracellular U73122 (gray circles, *N* = 6, *P* > 0.05) and AIP (black circles, *N* = 4, *P* > 0.05). **(B)** Same as **(A)**, but PNs were loaded with chelerytrine (5 μM in the pipette solution, left insets and gray circles, *N* = 5, P < 0.001) or H89 (10 μM, right insets and black circles, *N* = 5, P>0.05). **(C)** Summary data showing the EPSC peak amplitude under P_I_TX (50 μM, *N* = 10, *P* < 0.001) and effects of loading the PN with U73122 (5 μM, *N* = 6, *P* > 0.05), AIP (5 mg/ml, *N* = 4, *P* > 0.05), Chelerytine (5 μM, *N* = 5, *P* < 0.001) and H89 (10 μM, *N* = 6, *P* > 0.05), relative to EPSCs in control ACSF (100%). 60 EPSP-Ca^2+^ spikes were used in **(A–C)**.

Cytosolic Ca^2+^-mediated activation of intracellular kinases can induce LTP through an increase in the number of functional AMPARs in dendritic spines (Fernández de Sevilla et al., 2008). Kinases can also enhance NMDAR-mediated responses by changing the biophysical properties of NMDARs (Fernández de Sevilla and Buño, 2010). We therefore tested whether activation of the CaMKII was required to induce the LTP. Blockade of CaMKII with the peptide inhibitor AIP (5 μM in the pipette solution) suppressed LTP without preventing the EPSP-Ca^2+^ spike and ≈30 min after the induction process EPSCs reached values that were 93 ± 10% of the controls (*P* > 0.05; *N* = 4; Figures 6A,C).

We have shown that Ca^2+^ release from endoplasmic reticulum stores by activation of IP_3_Rs plays a key role in the cholinergic LTP in CA1 PNs (Fernández de Sevilla and Buño, 2010). The Ca^2+^ released plays a key role in long-term enhancement excitatory synaptic transmission (see above). Accordingly, we tested the effects of inhibiting the production of IP3 by blocking the PLC translocation with intracellular U73122. U73122 (2 mM in the pipette solution) inhibited the LTP without preventing the EPSP-Ca^2+^ spike and EPSCs reached values that were 97 ± 18% of the controls (*P* > 0.05; *N* = 6; Figures 6A,C). We next investigated the effects of inhibiting PKC with intracellular chelerytrine (5 μM); there was no effect on LTP and EPSCs reached values that were 191.47 ± 30% (*P* < 0.001; *N* = 5) of the controls ≈30 min after induction. In contrast, PKA blockade with H89 dihydrochloridre (10 μM in the pipette solution) had no effect on the EPSP-Ca^2+^ spike but did inhibit the LTP and ≈30 min after induction, EPSCs reached values that were 92.4 ± 4% of the controls (*P* > 0.05; *N* = 6; Figures 6B,C). The above results suggest that this LTP requires CaMKII, PLC, and PKA activation.

### Reducing the Ca^2+^ spike and the associated Ca^2+^ signal or the AP burst induced LTD instead of LTP

**Figure 7:**
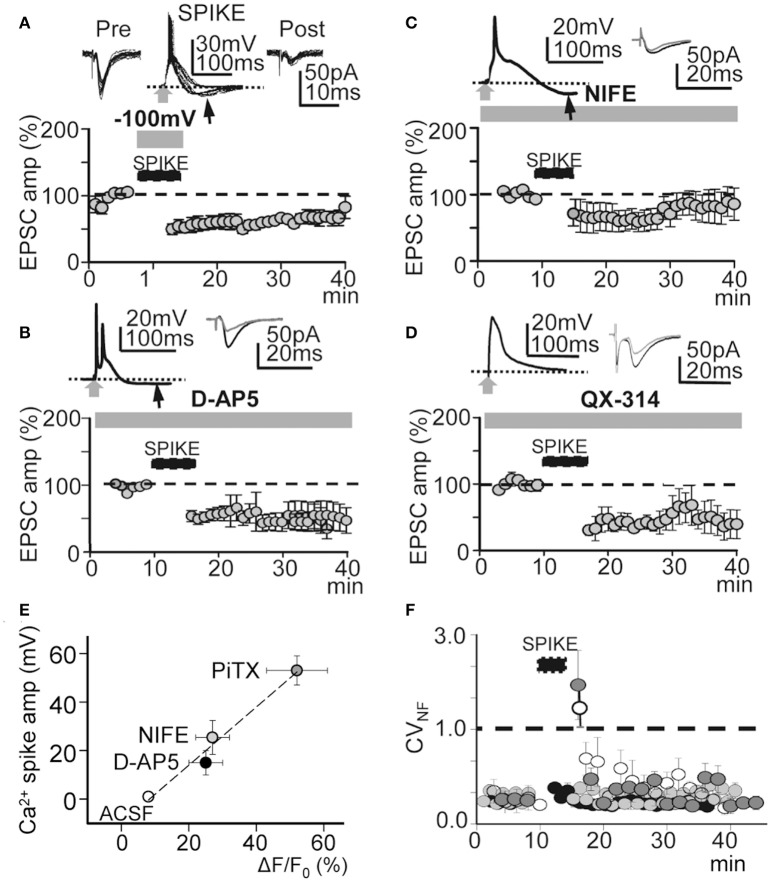
**Effects of hyperpolarization and NMDAR, L-type VGCC, and voltage-gated Na^**+**^ conductance blockade**. **(A)**, upper. Superimposed records of pre-induction EPSCs (*n* = 20), Ca^2+^ spikes during hyperpolarization to −100 mV (*n* = 20) and post-induction EPSCs (*n* = 10). **(A)**, lower. Same as Figure 4A, but LTD was induced with hyperpolarization to -100 mV during the INDUCTION process (*N* = 5, *P* < 0.001). **(B)** Same as **(A)**, but the LTD was induced under inhibition of NMDARs with D-AP5 (50 μM, *N* = 6, *P* < 0.001). **(C)** Same as **(A)**, but LTD induced under nifedipine (20 μM) inhibition of L-type VGCC (*N* = 5, *P* < 0.001). **(D)** Same as **(A)**, but the LTD was induced by intracellular QX-314 (5 mM in the pipette solution) inhibition of voltage gated Na^+^ conductance (*N* = 6, *P* < 0.001). **(E)** Plot of the Ca^2+^ spike amplitude in function of the cytosolic peak somatic Ca^2+^ signal recorded in control ACSF (open circle) and when PiTX (dark gray circle), nifedipine (gray circle) and D-AP5 (black circle) were added. Note the linear correlation (*r*^2^ = 0.98) indicating a close association between the amplitude of Ca^2+^ spikes the cytosolic Ca^2+^ signal. **(F)** Time course of the noise free coefficient of variation (CV_NF_) calculated form the experiments under intracellular QX-314 (white circles), nifedipine (dark gray circles), D-AP5 (light gray circles) and during hyperpolarization to −100 mV (black circles). Note the lack of long term modifications of the CV_NF_ in all conditions tested. Black arrows in **(A–C)** indicate hyperpolarizations following Ca^2+^ spikes.

Protocols that produce strong membrane depolarization induce LTP while those that cause modest or local depolarization generate LTD (Holthoff et al., 2004). This bidirectional behavior is thought to be caused by the level of Ca^2+^ influx during dendritic depolarization (Artola et al., 1990; Bliss and Collingridge, 1993; Neveu and Zucker, 1996; Dan and Poo, 2004; Kampa et al., 2007). Accordingly, we analyzed the effects of membrane hyperpolarization during the induction process. Hyperpolarization to −100 mV decreased the amplitude and duration of averaged EPSP-Ca^2+^ spike to 27 ± 2 mV and 50 ± 8 ms (*P* < 0.01; *N* = 5). In these conditions repeated basal stimulation (60 times at 0.2 Hz) induced an LTD that rapidly reached values of 63 ± 1% of the controls (*P* < 0.001; *N* = 5; Figure 7A). A NMDAR blockade with D-AP5 (50 μM) reduced the average amplitude and duration of Ca^2+^ spikes to 25 ± 4 mV and 55 ± 13 ms (*P* < 0.01; *N* = 5). In these conditions, basal stimulation (as above) produced a potent LTD that reached values that were 56 ± 3% of the controls (*P* < 0.01; *N* = 6) (Figure 7B). Nifedipine (20 μM) blockade of L-type VGCC also reduced the amplitude and duration of the Ca^2+^ spikes to 21 ± 1 mV and 100 ± 9 ms (*P* < 0.01; *N* = 5). Thirty min after induction, repeated basal stimulation (as above) induced a slowly declining LTD that reached values of 77 ± 5% of the controls (*P* < 0.05; *N* = 5; Figure 7C). A single or a pair of AP and Ca^2+^ spikes was followed by hyperpolarization in Figures 7A–C.

Moreover, a robust LTD that reached values of 48 ± 6% of the controls (*P* < 0.01; *N* = 6; Figures 7D, 4B) was induced when APs were inhibited by QX-314. Under QX-314 Ca^2+^ spikes were larger than those linked with the LTP (see above). These results suggest that what was required for LTP was not just the Ca^2+^ rise, but that Na^+^-mediated back-propagating APs played a role. In contrast, with the LTP that increased gradually in amplitude following induction, the LTD in these circumstances was fully developed at the end of the induction process.

The Ca^2+^ spike is strongly linked with Ca^2+^ influx, consequently an analysis of the relationship between the Ca^2+^ spike and the cytosolic Ca^2+^ signal could provide a direct estimate of the conditions that induce the bidirectional synaptic plasticity. Therefore, we recorded the somatic Ca^2+^ signals associated with the Ca^2+^ spike in control ACSF and under PiTX, PiTX + D-AP5, and PiTX + nifedipine. In control ACSF small Ca^2+^ signals with Δ*F/F*_*0*_ values of 8 ± 1% of the controls (*P* < 0.01; *N* = 6) were induced when APs were triggered in the absence of Ca^2+^ spikes. Under PiTX (50 μM) the Δ*F/F*_*0*_ reached much higher values that were 52 ± 9% of the controls (*P* < 0.001; *N* = 6), whereas lower values of 25 ± 5% (*P* < 0.00; same cells) were attained when D-AP5 (50 μM) was added to block NMDARs. Under nifedipine (50 mM) added to inhibit L-type VGCC, Δ*F/F*_*0*_ achieved values of 27 ± 5% from the controls (*P* < 0.001; *N* = 5) (Figure 7E). Taken together the above results suggest that the direction of the induced change in synaptic plasticity could be regulated by the degree of membrane depolarization and the cytosolic Ca^2+^ level attained during the EPSP-Ca^2+^ spike.

To verify the pre- or postsynaptic origin of this LTD we first tested for possible decreases in release probability at excitatory synapses. We plotted the noise-free coefficient of variation (CV_NF_) vs. time. Under all LTD-inducing manipulations, the CV_NF_ values remained below 1.0 (Figure 7F), suggesting the absence of significant changes in Glu release probability during an LTD and meaning that the synaptic depression originated postsynaptically.

### Bidirectional plasticity of whisker-evoked responses in anesthetized rats

**Figure 8:**
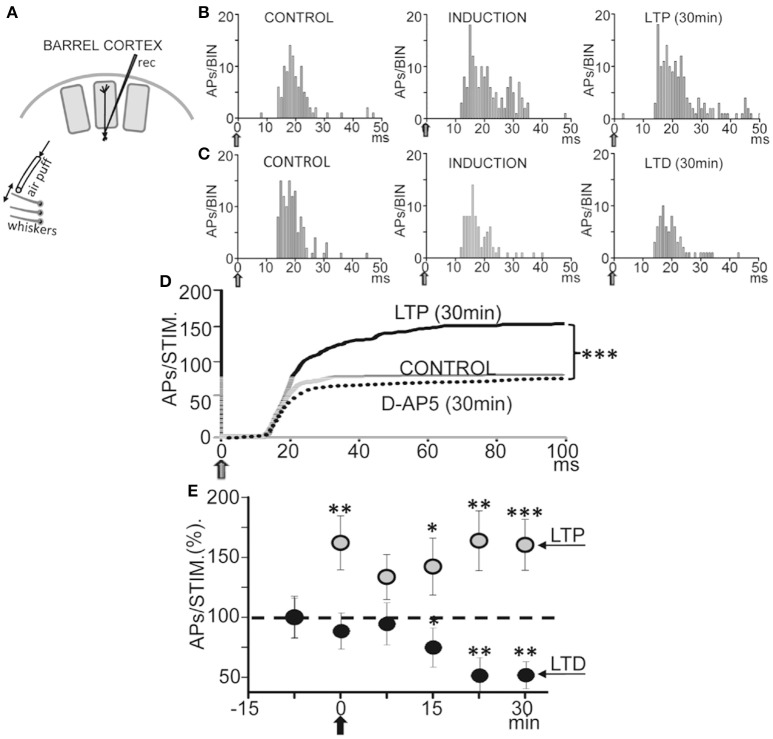
**Bidirectional plasticity evoked by whisker deflections in anesthetized rats. (A)** Schematic diagram of experimental setup. **(B)** Representative PSTHs computed during whisker deflections (gray arrows) in CONTROL (0.5 Hz), INDUCTION (1.0 Hz), and LTP (0.5 Hz) conditions. Note the increase in the number of APs during induction and the LTP 30 min after. **(C)** Same as **(B)**, but the number of APs was reduced 30 min after INDUCTION, or LTD, and APs did not increase during induction. **(D)** Cumulative spike plots (CSP) computed by averaging all PSTHs (see Materials and Methods) in CONTROL, LTP (*N* = 16, *P* < 0.001) and under an injection of D-AP5 (50 μM; 0.1 μl, *N* = 14, *P* < 0.001) in L5 (see Materials and Methods). Note the enhanced whisker-evoked response during LTP and the reduced response with D-AP5. Blockade of NMDARs by injections of D-AP5 inhibited plasticity. **(E)** Plot showing averaged responses vs. time before and after induction (black arrow). Data points represent the mean area of PSTHs (CSPs;% of controls) computed over 5 min showing LTP (gray circles, *N* = 16, *P* < 0.001) and LTD (black circles, *N* = 5, *P* < 0.01).

We first tested if repetitive low-frequency deflections delivered at the principal whisker (Figure 8A) could induce long-term response changes resembling those that occur *in vitro*. The 37 L5 neurons recorded were either silent or displayed a low spontaneous firing rate (0.5–2 APs/s), and responded to contralateral displacements of the principal whisker. Control whisker-evoked responses had on average 3.5 ± 0.5 APs/stimulus (measured from 0 to 100 ms after the stimulus onset). The low spontaneous AP firing rate and the activation by deflection of the principal whisker provide strong support to the notion that recordings were obtained from L5A PNs, as has been described previously (Manns et al., 2004; de Kock and Sakmann, 2009).

Following a 60 s control stimulation at 0.5 Hz a train of 40 stimuli at 1.0 Hz induced a long-lasting enhancement of the response (or LTP) from 3.9 ± 0.4 APs/stimulus in control to 5.3 ± 0.6 APs/stimulus, measured 30 min after induction in 16 neurons out of 23 (or 66%; *P* < 0.001; Figures 8B,D,E), while 5 neurons (22%) reduced their response (or LTD; from 3.5 ± 0.5 APs/stimulus in the control to 2.4 ± 0.1 APs/stimulus; *P* < 0.01; Figures 8C–E) and 2 neurons (9%) were not affected (from 3.2 ± 0.6 APs/stimulus in the control to 3.4 ± 0.4 APs/stimulus after induction; *P* > 0.05). Figure 8E shows the time course of the AP response plasticity.

### Different responses during induction typified cells that showed LTP or LTD

Interestingly, the whisker-evoked response was enhanced during the 1 Hz induction from 3.9 ± 0.41 APs/stimulus in the control to 5.5 ± 0.58 APs/stimulus (*P* < 0.01; *N* = 5) in neurons that showed LTP (Figure 8B). In contrast, in neurons that showed LTD whisker-evoked responses were not altered during induction (from 3.5 ± 0.48 APs/stimulus in control to 2.9 ± 0.37 APs/stimulus; *P* > 0.05; *N* = 5; Figure 8C).

### Bidirectional plasticity required NMDAR activation

We next tested if blockade of NMDARs by injection of D-AP5 (50 μM; 0.1 μl) in L5 (see Section Materials and Methods) prevented plasticity. To exclude possible artifacts caused by this manipulation we checked that the D-AP5 injection did not modify AP amplitudes. Under the effects of D-AP5 the whisker-evoked response measured ≈30 min after the 1 Hz induction stimulation train was essentially identically to the control response and plasticity was absent (from 2.6 ± 0.38 in control to 2.5 ± 0.36 APs/stimulus after induction; *P* > 0.05; *N* = 14; Figure 8D). Therefore, the above results suggest that the activation of NMDA receptors in L5 play a key role in the genesis of the bidirectional synaptic plasticity.

## Discussion

Here, we describe a form of bidirectional plasticity induced by unpaired low-frequency stimulation of basal inputs in regular spiking L5 PNs of the rat barrel cortex. This stimulation can trigger an EPSP closely followed by an AP burst and Ca^2+^ spike that were present when the GABA_A_Rs were blocked with P_I_TX. In contrast, Ca^2+^ spikes and LTP were absent when synaptic inhibition was active, revealing a powerful GABA_A_ inhibitory control of excitability and synaptic plasticity (Kampa et al., 2007; Caporale and Dan, 2008; Sjöström et al., 2008; Feldman, 2012; Hao and Oertner, 2012; Chiu et al., 2013; Hsieh and Levine, 2013).

This LTP (present Results) has all the attributes of an activity-dependent hebbian LTP. Indeed, it required: (*i*) activation of NMDARs; (*ii*) depolarization to facilitate Ca^2+^ influx through NMDARs; and (*iii*) AP backpropagation facilitated by the a dendritic Ca^2+^ spike. NMDARs are thought to represent the “coincidence detector” that links synaptic input with postsynaptic depolarization. Depolarization is required to relieve the extracellular voltage-dependent Mg^2+^ block of the NMDA channel and allow Ca^2+^ influx (Schiller and Schiller, 2001; Kampa et al., 2004, 2007; Fuenzalida et al., 2010). Therefore, the EPSP-Ca^2+^ spike fulfills the attributes of a coincidence detector because it associates the EPSP with the Ca^2+^ influx through NMDARs, a Ca^2+^ influx that is facilitated by the depolarization contributed by the activation of L-type VGCC. In addition, the resulting depolarization triggers backpropagation of the AP burst that is a prerequisite for the induction of LTP in L5 PNs (Larkum et al., 1999a; Kampa et al., 2004).

The EPSP-Ca^2+^ spike fulfills the components that define STDP and represents a simple form of the hebbian LTP induction protocol, one that obeys the original hebbian rule (Hebb, 1949). We also show that the same presynaptic stimulation can induce LTD when the Ca^2+^ spike and the associated cytosolic Ca^2+^ signal were reduced or when the AP burst is inhibited. The EPSP-AP STDP protocol is ineffective in L5 PNs because single APs are not back-propagated (Stuart et al., 1997; Kampa and Stuart, 2006). However, AP bursts evoked by depolarizing current injection can trigger Ca^2+^ spikes that boost AP backpropagation and induce LTP by STDP in L5 PNs (Larkum et al., 1999a; Kampa et al., 2004). The AP burst associated with Ca^2+^ spikes, (present Results) and the EPSP-AP burst triggered by the postsynaptic current injection of Larkum et al. (1999b) and Kampa and Stuart (2006), can be considered to accomplish an essentially identical operational role that facilitates AP backpropagation and produces a robust dendritic Ca^2+^ signal that induces LTP (present Results). In addition, we show that in anesthetized rats equivalent bidirectional plasticity is induced in L5 neurons when whiskers are repeatedly deflected.

We used higher stimulation intensities to trigger EPSP-Ca^2+^ spikes during the induction process than the intensities used to evoke control EPSCs. The larger EPSPs evoked by the additional Glu released by presynaptic fibers recruited by the higher stimulation intensity was able to relieve the Mg^2+^ blockade of NMDA receptors at the weakly-stimulated synapses and potentiate those synapses. However, heterosynaptic input did not appear to contribute to this LTP because high intensity induction protocols were ineffective when the AP bursts or the Ca^2+^ spikes were blocked.

Inducing the LTP required stimulation within a narrow repetition rate. The stimulation rate used here (0.2–0.3 Hz) approximately matches the slow firing frequencies of cortico-thalamic circuits (Steriade et al., 1993) and of a subset of L5 PNs (Lorincz et al., 2015) during specific behavioral states. Therefore, this LTP could be facilitated during these states.

With classic STDP protocols the degree and sign of the synaptic change is critically dependent on the timing between the EPSP and the postsynaptic spikes (Bi and Poo, 1998; Fuenzalida et al., 2007). Although the time window is fixed by the EPSP-Ca^2+^ spike, the direction of the synaptic modification can be changed since LTD is induced when the Ca^2+^ spike and the associated cytosolic Ca^2+^ signal was reduced by hyperpolarization and when NMDARs or L-type VGCCs are blocked (present Results). Different levels of cytosolic Ca^2+^ are thought to control the magnitude and nature of the induced synaptic change by activating different molecular cascades (Lisman, 1989; Artola et al., 1990; Bliss and Collingridge, 1993; Cho et al., 2001; Kampa et al., 2007). STDP-induced LTP requires the sequential activation of NMDARs and VGCC within dendritic spines (Tigaret et al., 2016), as is likely to occur in these experiments.

Our present results show that the simple induction form of LTP studied here requires an EPSP followed by an AP burst and a robust dendritic Ca^2+^ spike mediated by activation of both NMDA and L-Type VGCC, and agrees with a previous report (Tigaret et al., 2016). Consequently, the degree of inhibition can control the type of synaptic change by regulating the magnitude of the Ca^2+^ spike. We showed that, with intact inhibition under superfusion with acetylcholine (ACh), low-frequency basal stimulation can evoke the EPSP-Ca^2+^ spike and LTP in L5 PNs by enhancing EPSPs and reducing IPSPs through activation of AChRs (Nuñez et al., 2012). Consequently, low-frequency stimulation of basal inputs could theoretically induce LTP under natural physiological conditions when mAChRs are activated or the activity of inhibitory interneurons is decreased or GABA release probability is reduced through activation of type 1 endocannabinoid receptors (CB_1_Rs); these attractive possibilities remain to be investigated. A novel form of plasticity has recently been reported in tuft dendrites of L5 PNs (Sandler et al., 2016). This plasticity is induced by unpaired low-frequency (0.1 Hz) stimulation of the tuft inputs and requires Kv4.2 channels, NMDARs, membrane internalization and AMPAR insertion. It is different from the plasticity reported here, but nevertheless it also required GABA_A_R blockade. These two forms of plasticity at separate processing and storing compartments in L5 PNs can possibly be coupled or uncoupled by the dynamic regulation of dendritic excitability. Therefore, they could either function in site-specific or in a cooperative manner depending on system demands.

The LTP reported here required activation of mGluR1, CaMKII, G-proteins, PLC and PKA. Postsynaptic group I mGluRs are crucial for the induction of LTP because they lead to depolarization, increased excitability and LTP at glutamatergic synapses (Lisman et al., 2002; Lamsa et al., 2007). Neocortical expression of hebbian LTP requires CaMKII activation (Otmakhov et al., 1997; Malenka and Nicoll, 1999; Fukunaga and Miyamoto, 2000). In addition, mAChR activation can induce LTP in both the hippocampus and the barrel cortex where release from IP_3_-sensitive intracellular Ca^2+^stores plays a key role (Rose and Konnerth, 2001; Fitzjohn and Collingridge, 2002; Fernández de Sevilla et al., 2008; Fernández de Sevilla and Buño, 2010; Baker et al., 2013; Domínguez et al., 2014). Moreover, nAChRs can enhance excitatory and reduce inhibitory synaptic transmission (Buccafusco et al., 2005; Nuñez et al., 2012; Udakis et al., 2016). Thus, equivalent signaling cascades activated through different mechanisms can lead to similar long-term synaptic modifications.

We show that equivalent bidirectional plasticity is induced in L5 neurons when whiskers are repeatedly deflected at 1.0 Hz. Interestingly, this plasticity mainly consists in a modification of the late component of the whisker-evoked response and is dependent on the activation of NMDARs. Rats exploring the environment move their whiskers on objects or surfaces in repeated rhythmic sweeps at frequencies of 4–12 Hz (Carvell and Simons, 1990; Fanselow and Nicolelis, 1999). In contrast, resting rats either do not move their whiskers or do so at low-frequencies <1 Hz. It has been shown that repetitive whisker deflections at the frequency used to explore the environment induce a long-lasting response facilitation of cortical neurons by activation of NMDA receptors (Barros-Zulaica et al., 2014). We now show that low-frequency stimulation can induce bidirectional plasticity of cortical barrel neurons through activation of NMDARs. Spontaneous ACh release is lower under urethane anesthesia than in freely moving animals (Bertorelli et al., 1991), but basal ACh release is sustained in both conditions (Rasmusson et al., 1992; Jiménez-Capdeville et al., 1997). Cortical ACh reduces GABAergic transmission (Nuñez et al., 2012), this may explain why LTP is induced *in vivo*, while LTP required GABA_A_R blockade *in vitro*. Therefore, it is likely that this form of bidirectional plasticity that is present both *in vivo* and *in vitro* under the inhibitory regulation of dendritic excitability could control the flow and storage of select input characteristics and regulate behavior and the flow of sensorimotor information in natural conditions. Importantly, Ca^2+^ activity in the apical dendrites and AP bursts in L5 PNs in mice are correlated with the threshold for perceptual detection of whisker deflections (Takahashi et al., 2016), demonstrating that active dendritic mechanisms are causally linked to perceptual detection and behavior.

## Author contributions

AD, DF, and NB performed the experiments; DF, AN, and WB designed the experiments; AD, NB, DF, AN, and WB analyzed data; AD, NB, DF, AN, and WB wrote the manuscript and edited and approved the final version.

### Conflict of interest statement

The authors declare that the research was conducted in the absence of any commercial or financial relationships that could be construed as a potential conflict of interest.
