# Corrigendum: Bidirectional Hebbian Plasticity Induced by Low-Frequency Stimulation in Basal Dendrites of Rat Barrel Cortex Layer 5 Pyramidal Neurons

**DOI:** 10.3389/fncel.2017.00112

**Published:** 2017-05-03

**Authors:** Andrea Díez-García, Natali Barros-Zulaica, Ángel Núñez, Washington Buño, David Fernández de Sevilla

**Affiliations:** ^1^Departamento de Anatomía, Histología y Neurociencia, Facultad de Medicina, Universidad Autónoma de MadridMadrid, Spain; ^2^Consejo Superior de Investigaciones Científicas, Instituto CajalMadrid, Spain

**Keywords:** Ca^2+^ spikes, NMDARs, L-type VGCC, dentritic excitabilty, STDP

There is an error in the Acknowledgments section. The correct number for BFU2016-80802-P is BFU2016-80802-P AEI/FEDER, UE. The correct name for the Funder is Ministerio de Ciencia y Tecnología, Ministerio de Ciencia e Innovación, and Ministerio de Economia y Competitividad.

The authors apologize for this error and state that this does not change the scientific conclusions of the article in any way.

## Conflict of interest statement

The authors declare that the research was conducted in the absence of any commercial or financial relationships that could be construed as a potential conflict of interest.

